# Dual Infection and Superinfection Inhibition of Epithelial Skin Cells by Two Alphaherpesviruses Co-Occur in the Natural Host

**DOI:** 10.1371/journal.pone.0037428

**Published:** 2012-05-21

**Authors:** Keith W. Jarosinski

**Affiliations:** Department of Microbiology and Immunology, Cornell University, Ithaca, New York, United States of America; Queen’s University, Canada

## Abstract

Hosts can be infected with multiple herpesviruses, known as superinfection*;* however, superinfection of cells is rare due to the phenomenon known as superinfection inhibition. It is believed that dual infection of cells occurs in nature, based on studies examining genetic exchange between homologous alphaherpesviruses in the host, but to date, this has not been directly shown in a natural model. In this report, *gallid herpesvirus 2* (*GaHV-2*), better known as Marek’s disease virus (MDV), was used in its natural host, the chicken, to determine whether two homologous alphaherpesviruses can infect the same cells *in vivo*. MDV shares close similarities with the human alphaherpesvirus, varicella zoster virus (VZV), with respect to replication in the skin and exit from the host. Recombinant MDVs were generated that express either the enhanced GFP (eGFP) or monomeric RFP (mRFP) fused to the UL47 (VP13/14) herpesvirus tegument protein. These viruses exhibited no alteration in pathogenic potential and expressed abundant UL47-eGFP or -mRFP in feather follicle epithelial cells *in vivo*. Using laser scanning confocal microscopy, it was evident that these two similar, but distinguishable, viruses were able to replicate within the same cells of their natural host. Evidence of superinfection inhibition was also observed. These results have important implications for two reasons. First, these results show that during natural infection, both dual infection of cells and superinfection inhibition can co-occur at the cellular level. Secondly, vaccination against MDV with homologous alphaherpesvirus like attenuated *GaHV-2,* or non-oncogenic *GaHV-3* or *meleagrid herpesvirus* (*MeHV-1*) has driven the virus to greater virulence and these results implicate the potential for genetic exchange between homologous avian alphaherpesviruses that could drive increased virulence. Because the live attenuated varicella vaccine is currently being administered to children, who in turn could be superinfected by wild-type VZV, this could potentiate recombination events of VZV as well.

## Introduction

Marek’s disease (MD) is caused by *gallid herpesvirus 2* (*GaHV-2*), better known as MD virus (MDV). MDV is a member of the *Mardivirus* genus in the subfamily of *Alphaherpesvirinae* [1]. Symptoms of MD include immune suppression, neurologic signs such as paralysis and ataxia, and the development of lymphoproliferative disease in chickens characterized by solid tumors in the viscera and other organs. Natural infection begins through inhalation of virus, after which MDV is taken to the lymphoid organs and primary cytolytic replication in B and then T lymphocytes ensues [2], [3]. Following lytic infection, latency is established mainly in activated CD4^+^ T cells, which may be transformed into highly proliferative T cell lymphomas, depending on the genotype of the infected chicken and strain of virus. Irrespective of the transformation event, infection of feather follicle epithelial (FFE) cells in the skin by migrating infected lymphocytes leads to the production of infectious particles that are shed into the environment, providing a continuous source of infectious virus. The lifecycle of MDV is similar to a human alphaherpesvirus, varicella zoster virus (VZV, *human herpesvirus 3*, *HHV-3*), that causes varicella, commonly called chicken pox, during primary infection and herpes zoster, commonly referred to as shingles, during reactivation from latency. Both viruses enter the host through the respiratory tract, initially infect epithelial cells and then lymphocytes, which transport virus to the skin where infectious virus is produced in epithelial skin cells and shed into the environment [2], [4].

Intraspecific recombination of alphaherpesviruses, that is, recombination between alphaherpesviruses within the same subfamily, occurs frequently *in vivo* [5]. It has been documented that inoculation of two attenuated or mutant strains of alphaherpesviruses into animals can result in production of virulent virus, as has been shown for herpes simplex virus 1 (HSV-1, *human herpesvirus 1*, *HHV-1*) [6], [7], pseudorabies virus (PRV, *suid herpesvirus 1*, *SuHV-1*) [8]–[14], *bovine herpesvirus 1* (*BoHV-1*) [15], and infectious laryngotracheitis virus (ILTV, *gallid herpesvirus 1*, *GaHV-1*) [16]. Intraspecific recombination is dependent on at least three requisites. First, there has to be significant homology between the two viruses, at the genetic level, so that recombination could occur. Second, both viruses need to infect the same host and third, both viruses need to infect the same cell for recombination to proceed. Without this, recombination between two viruses would be highly unlikely. It has been shown that dual infection of cells by PRV occurs within the animal. Banfield *et al.* showed that two attenuated PRV strains, one expressing enhanced GFP (eGFP) and the other expressing monomeric RFP (mRFP), could infect the same neurons when injected into the anterior chambers of separate rat eyes [17]. Dual infection of PRV in rat neurons was also recently shown using mutant viruses expressing fluorescent proteins [18]. To date, there are no published studies directly analyzing dual infection of cells by two homologous alphaherpesviruses within a natural host.

Superinfection inhibition, whereby infection of a cell by one virus inhibits dual infection by a second virus, has been described for many viruses of bacteria, plants, and animals [19]–[28]. Banfield *et al.* used primary cultures of rat dorsal root ganglia (DRG) and showed that dual infection of cells with two PRVs occurred with high frequency (∼100%) when the primary rat DRG cultures were infected simultaneously (coinfection) with both viruses [17]. When one virus was inoculated followed by the second (superinfection) ≥4 h later, dual infection of the DRG was very infrequent (∼1%). Thus, their data strongly suggested a significant amount of superinfection inhibition occurs during infection of primary rat DRG neurons *in vitro*. To date, it has not been demonstrated that superinfection inhibition occurred *in vivo* during infection with homologous herpesviruses.

In the case of MDV, dual infection of cells by two different viruses is of particular importance because since the 1960’s, MD has largely been controlled using homologous avirulent vaccines. These vaccines generally prevent the development of MDV-induced tumors and disease, but do not prevent superinfection with pathogenic MDV [29]. Non-oncogenic turkey herpesvirus (HVT, *meleagrid herpesvirus 1*, *MeHV-1)*, non-oncogenic chicken herpesvirus 3 (*gallid herpesvirus 3*, *GaHV-3*), and attenuated MDV (att*GaHV-2*) have been used during the last four decades in vaccination programs against MD. Modified live vaccines are typically administered to newly hatched chicks or *in ovo* at 18 to 19 days of embryonation, but are exposed to challenge virus almost immediately in commercial settings [30]. It is widely accepted that the use of highly homologous vaccines against MD have ultimately led to increasing virulence of pathogenic MDV strains [31]. The long history of non-sterilizing immunity induced by MD vaccines, the increasing virulence of MDV due to vaccination, and prior evidence of intraspecific genetic exchange within strains of the HSV, PRV, BoHV-1, and ILTV alphaherpesviruses in the host are troublesome. Thus far, there is no evidence showing that exchange of genetic material between wild-type MDV and MD-vaccine strains occurs in nature; however, this has been mostly due to a limitation in the molecular tools needed for such studies. We now have efficient tools to generate virulent and attenuated recombinant (r)MDV that express fluorescent proteins for visual detection *in vivo,* without leaving genetic scars in the genome or altering pathogenesis, and importantly, a natural alphaherpesvirus-host model in which the results are direct and not dependent on the use of “host-adapted” strains that can complicate interpretation of data. The question of whether cells can be dually infected during natural alphaherpesvirus infection is significant since the recent introduction of the first effective vaccine against a human alphaherpesvirus, VZV [32]. The long history of non-sterilizing vaccination against MD in chickens, taken together with the similar virus life cycles that MDV and VZV use to enter and exit the host, highlight the importance of understanding whether homologous viruses can regularly infect the same cells in a natural host.

A two-step Red-mediated recombination strategy was utilized in which the UL47 (VP13/14) tegument protein of MDV was tagged with either eGFP or mRFP in virulent and attenuated rMDVs. These rMDVs were generated to determine, through direct visual examination, whether two similar, but distinguishable, alphaherpesviruses can dually infect the same cells in the animal. For clarity, the term “coinfection” is used in this report to describe simultaneous infection of chickens with two rMDVs, “superinfection” to describe infection of chickens with two rMDVs at different times (7 and 14 days between inoculations), and “dual infection” to describe infection of individual cells with two rMDVs. In two superinfection experiments performed, replication of the second virus within FFE cells of chickens was rarely observed. However, in coinfected chickens, dual infection and replication of both viruses was clearly observed in individual cells, irrespective of the virulence of the virus. There was also visual evidence that strongly suggested superinfection inhibition occurs at the cellular level in the host. These data conclusively show that two similar alphaherpesviruses can infect the same cells *in vivo*, potentiating the exchange of genetic material, while also showing that superinfection inhibition can co-occur. How these two, seemingly opposing events, can co-occur is discussed.

## Results

### Generation and *in vitro* Growth of vUL47-eGFP and -mRFP

**Figure 1:**
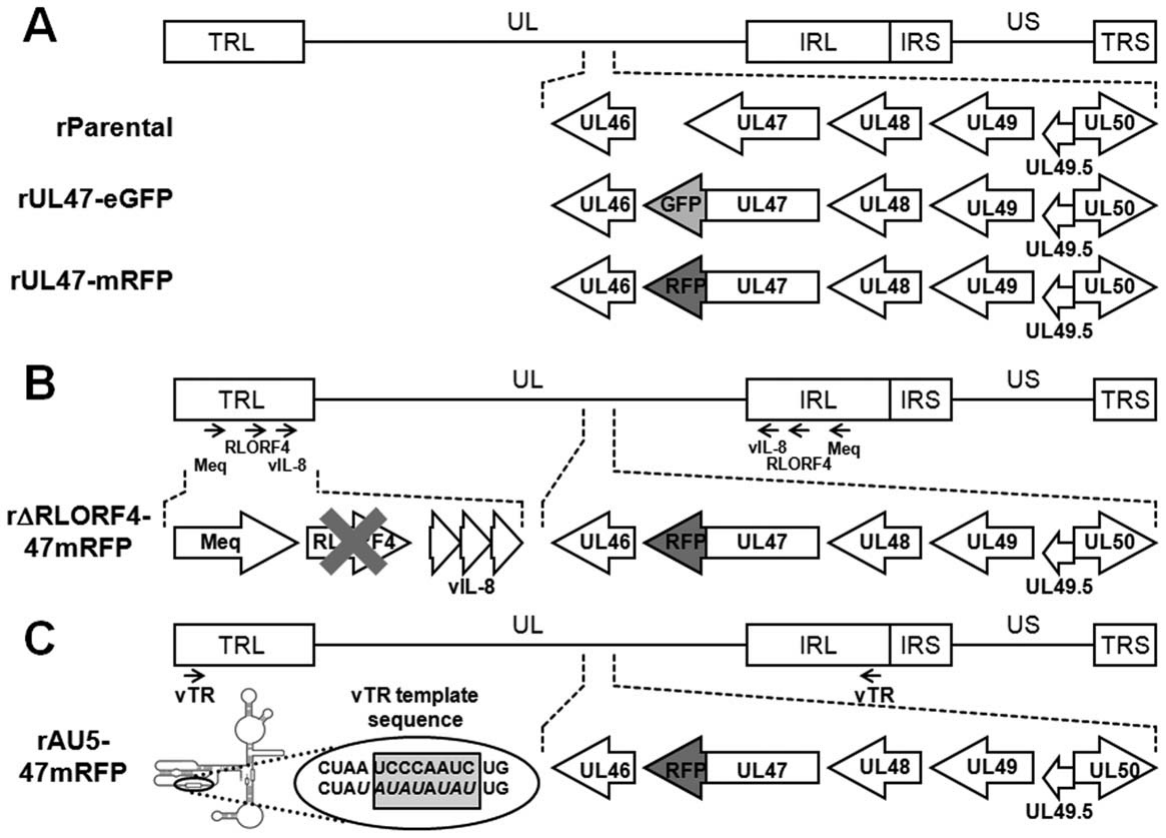
Generation of UL47-eGFP and UL47-mRFP fusion proteins in rMDV. Shown for each clone is the MDV genome depicting the locations of the terminal repeat long (TRL) and short (TRS), internal repeat long (IRL) and short (IRS), and unique long (UL) and short (US) regions. The position and orientation of the UL47 gene with respect to adjacent genes within the UL are shown. The fluorescent proteins eGFP and mRFP were fused to the C terminus of the UL47 tegument protein in four different BAC clones to generate rUL47-eGFP and rUL47-mRFP (A), rΔRLORF4-47mRFP (B), and rAU5-47mRFP (C) using the previously described BAC clones for rRB-1B [51], rRB-1B-ΔRLOR4 [36], and rAU5 vTR [37]. Only genes within the TRL are shown in the figure for simplicity, though the second copy of each gene within the IRL was also altered so that both copies of RLORF4 were deleted or the template sequence of both vTR copies were mutated in each clone.

Recently, fully virulent fluorescent rMDV was generated by fusing eGFP to the C terminus of the UL47 (VP13/14) tegument protein [33]. This virus showed no reduced pathogenicity and expression of the UL47-eGFP protein was abundant in the FFE cells in the skin during *in vivo* infection. This strategy was utilized to generate a red virus that could be distinguished from the green virus in coinfection and superinfection studies. To do this, mRFP was fused to the C terminus of the UL47 in three rMDV clones using previously described Red recombination techniques [34], [35]. Figure 1 shows a schematic representation of the rMDVs generated for this report and Table 1 shows the history of each virus. To generate fully virulent virus, mRFP was fused to the C terminus of UL47 in the parental clone (rUL47-mRFP), as was done previously with eGFP (rUL47-eGFP). Also generated were two viruses previously characterized as attenuated rMDV due the deletion of both copies of RLORF4 [36] or mutation of the viral telomerase RNA (vTR) template sequence [37]. Both mutant viruses were found to replicate in chickens, but at reduced capacity compared to the highly virulent parental viruses.

**Table 1:** Viruses used in this report.

Name^a^	Originating clone (ref)^b^	Overall Modifications (reference)^c^
vParental	pRB-1B (1232) [51]	None
vUL47-eGFP	pRB-1B (1232) [51]	eGFP fused to C terminus of UL47 [33]
vUL47-mRFP	pRB-1B (1232) [51]	mRFP fused to C terminus of UL47
vΔRLORF4	pRB-1B (1232) [51]	Both copies of RLORF4 deleted [36]
vΔRLORF4-47mRFP	ΔRLORF4 in pRB-1B [36]	Both copies of RLORF4 deleted and mRFP fused to the C terminus of UL47
vAU5 vTR	pRB-1B (1232) [51]	Both vTR template sequences mutated [37]
vAU5-47mRFP	vTR AU5mut in pRB-1B [37]	Both vTR template sequences mutated and mRFP fused to the C terminus of UL47

a Designated name of virus.

b Originating bacterial artificial chromosome clone used in which the fluorescent protein was fused to UL47 and its original publication.

c Final history of modifications in the recombinant virus and the reference for viruses previously used.

Following the generation of the rMDV bacterial artificial chromosome (BAC) clones and reconstitution of virus in primary chicken cells, the *in vitro* growth properties of the rMDVs were evaluated using plaque area assays. As was previously shown for vUL47-eGFP [33], vUL47-mRFP replicated in a manner indistinguishable from vParental (Fig. S1). Deletion of both copies of RLORF4 (vΔRLORF4) induced significantly greater plaque areas compared to vParental, consistent with previous results [36], and fusing mRFP to UL47 (vΔRLORF4-47mRFP) did not affect this. vAU5 and vAU5-47mRFP generated plaques similar to vParental, which was expected from previous results [37]. These data show that fusing mRFP to the UL47 tegument protein did not affect *in vitro* growth of rMDVs, similar to the UL47-eGFP fusion protein.

### *In vitro* Expression of UL47-eGFP and UL47-mRFP in rMDV

Next, reconstituted viruses were evaluated for UL47-eGFP and UL47-mRFP expression in chicken kidney cells (CKC) cultures. Figure S2 shows expression of UL47-eGFP in vUL47-eGFP and UL47-mRFP in vUL47-mRFP, vΔRLORF4-47mRFP, and vAU5-47mRFP in contrast to overall MDV protein expression. As was previously described during the generation of vUL47-eGFP [33], expression of UL47-mRFP was low in most cells infected with only a few select cells expressing high levels of UL47-mRFP (Fig. S2). The parental virus (vParental) had no detectable UL47-eGFP or UL47-mRFP.

### Pathogenicity of rMDVs Expressing UL47-eGFP or -mRFP

Chickens (n = 18 to 20 per group) were inoculated with vUL47-eGFP, vUL47-mRFP, vΔRLORF4-47mRFP, or vAU5-47mRFP at 7 days of age and evaluated for 8 weeks for signs of clinical MD, mainly characterized by wasting, paralysis, and gross T cell tumors, from 3 to 7 weeks post-infection (p.i.). vUL47-eGFP had previously been tested for disease induction [33]. In >90% of animals infected with vUL47-eGFP or vUL47-mRFP, clinical signs of MD developed, and following gross examination, tumors were observed and the percent tumor incidence determined (Fig. S3). Consistent with previous reports, deletion of RLORF4 led to severely reduced virulence [36] and mutation of the template area of vTR was completely non-oncogenic [37]. These results confirm that fusing mRFP to UL47 did not alter each rMDVs’ pathogenesis *in vivo* and that both ΔRLORF4-47mRFP and vAU5-47mRFP maintained their attenuated characteristics.

### Confirmation of UL47-eGFP and -mRFP in FFE Cells of the Chicken

**Figure 2:**
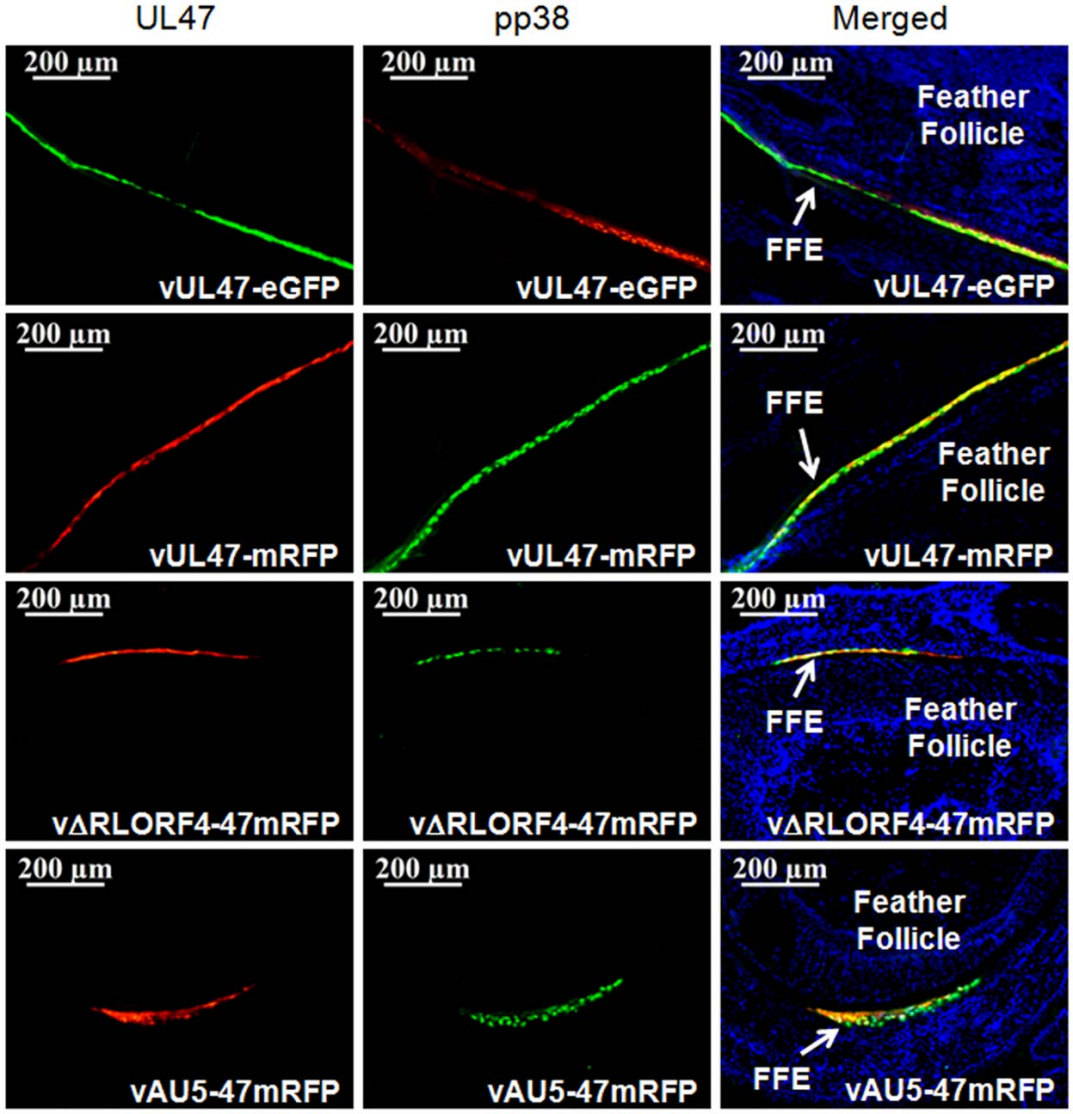
Expression of UL47-eGFP and UL47-mRFP by rMDVs *in vivo*. Skin/feather tissues were collected from vUL47-eGFP-, vUL47-mRFP-, vΔRLORF4-47mRFP, and vAU5-47mRFP-infected chickens at 21 days p.i. Tissues were sectioned transversely through the feather follicle and FFE cells infected with rMDVs were stained for the pp38 early lytic MDV protein and nuclei (blue) as described in the materials and methods and examined using the Axio Imager M1 system at ×200. Some cells can be seen that express only pp38 or UL47-eGFP/mRFP, while others express both (merging green and red  =  yellow). Feather follicles (FF) and FFE cells are indicated.

Figure 2 shows representative feather follicles infected with rMDVs expressing fluorescent proteins 21 days p.i. All four rMDVs expressed abundant UL47-eGFP or –mRFP in FFE cells examined, relative to the early lytic protein pp38 that is expressed predominantly during lytic replication [38]. Consistent with previous work, differential expression of the late UL47-eGFP or UL47-mRFP compared to early lytic pp38 was observed [33]. These results confirm the abundant expression of UL47 in FFE cells *in vivo*, regardless of the fluorescent protein fused to its C terminus.

### Dual Infection of FFE Cells During Coinfection with Two Highly Virulent rMDV

To address the question of whether two highly virulent rMDVs can infect the same FFE cells, chickens were inoculated with an equal titer [1,000 plaque-forming units (PFU) each] of vUL47-eGFP and -mRFP (n = 4), or 2,000 PFU of vUL47-eGFP (n = 2) or 2,000 PFU of vUL47-mRFP (n = 2) individually, in 7 day old chickens (Fig. S4A). Twenty-eight days p.i., the feather follicles were examined for replication of each virus using fluorescence microscopy.

The overall number of infected follicles and areas in which coinfection of feather follicles and dual infection of FFE cells were observed are summarized in Table 2. In chickens infected with each virus individually, 40.9% and 50.0% of the follicles examined were positive for UL47-eGFP and -mRFP, respectively. In the four coinfected chickens, all chickens were positive for infected follicles; however, only vUL47-mRFP appeared to be present in feather follicles of one of these birds. Intriguingly, one chicken had three follicles positive for vUL47-eGFP, nine follicles positive for vUL47-mRFP, and two areas within two follicles that appeared to be dually infected with both viruses (see below).

**Table 2:** Coinfection with virulent viruses (Experiment 1).

Group^a^	Analysis (days p.i.)^b^	Follicles (n)^c^	Green^d^	Red^e^	Coinfection follicle^f^	Dual infection cell^g^
						
vUL47-eGFP	28	10	3	0	0	0
		12	6	0	0	0
		% Infected:	40.9%	0.0%	0.0%	Total: 0
						
vUL47-mRFP	28	8	0	7	0	0
		12	0	3	0	0
		% Infected:	0.0%	50.0%	0.0%	Total: 0
						
vUL47-eGFP +vUL47-mRFP	28	10	3	9	2	1
		9	3	6	0	0
		12	0	3	0	0
		10	4	5	0	0
		% Infected:	24.4%	56.1%	4.9%	Total: 1
						

a Chickens were inoculated with 2,000 PFU of vUL47-eGFP or vUL47-mRFP, or with a mixture of 1,000 PFU of vUL47-eGFP and vUL47-mRFP each at 7 days of age.

b Skin samples were collected at 28 days post-inoculation (p.i.).

c The number of follicles examined for each chicken.

d The number of follicles positive for green fluorescence (vUL47-eGFP replication) and percent follicles infected per group.

e The number of follicles positive for red fluorescence (vUL47-mRFP replication) and percent follicles infected per group.

f The number of follicles positive for both green and red fluorescence and percent follicles infected with both viruses per group.

g The number of regions within follicles that were positive for both green and red fluorescence and the total per group.

Next, dual infection was examined during superinfection with two virulent rMDVs. In this experiment (Fig. S4B), twenty 7 day old chickens were inoculated with 2,000 PFU of vUL47-mRFP and after 7 or 14 days, ten of those chickens were inoculated with an equal titer of vUL47-eGFP. An additional chicken was inoculated with only vUL47-eGFP as a control. At 21 and 28 days after the first inoculation, skins were collected from five chickens for each group and infection of FFE cells with each virus was evaluated using fluorescence microscopy. Interestingly, for all superinfected chickens examined, only expression of UL47-mRFP could be observed, indicating that only vUL47-mRFP was effectively replicating in the feather follicles (Table S1). As a positive control for vUL47-eGFP replication, the control chicken inoculated with only this virus had 37.5% follicles positive for vUL47-eGFP replication, indicating the fitness of vUL47-eGFP could not explain its lack of replication in FFE cells in superinfected chickens.

### Dual Infection of FFE Cells During Coinfection with Attenuated and Virulent rMDV

Next, attenuated and virulent rMDVs were used to examine dual infection of cells during coinfection and superinfection of chickens. For superinfection, chickens were inoculated with 2,000 PFU of vΔRLORF4-47mRFP or vAU5-47mRFP at 7 days of age, and then superinfected with 2,000 PFU of virulent vUL47-eGFP 7 or 14 days later (Fig. S4C). Replication of each virus in FFE cells was then examined at 7, 14, or 21 days after the second inoculation. Again, during superinfection, only the first inoculated virus could be observed replicating in the feather follicles examined in almost all of the chickens (Table S2). In one chicken infected with vAU5-47mRFP then superinfected with vUL47-eGFP, only vUL47-eGFP was evident in the follicles tested. Interestingly, even in chickens with little to no active replication in the FFE cells of attenuated vAU5-47mRFP, superinfection with vUL47-eGFP was not seen. The control chicken inoculated with only vUL47-eGFP was positive for virus replication in 59.2% of the follicle again confirming the lack of vUL47-eGFP replication in FFE cells of superinfected chickens was not due to the fitness of the virus.

In a fourth experiment, dual infection was evaluated during coinfection with attenuated and virulent rMDVs (Fig. S4D). In this experiment, vAU5-47mRFP was not used since few follicles appeared to be infected in the previous experiment. In chickens infected with both vΔRLORF4-47mRFP and vUL47-eGFP, coinfection of feather follicles and dual infection of FFE cells was more readily apparent than in the first experiment. Table 3 summarizes these results. At 21 days after the second inoculation, 10.0% of follicles examined were coinfected and of those coinfected follicles, a total of six areas were clearly infected with both viruses. After 28 days following the second inoculation, 22.1% of follicles were coinfected and dual infection of FFE cells was also clearly observed.

**Table 3:** Coinfection with attenuated and virulent viruses (Experiment 4).

Group^a^	Analysis (days p.i.)^b^	Follicles (n)^c^		Green^d^		Red*^e^*		Coinfection Follicle*^f^*		Dual infection Cell*^g^*	
vΔRLORF4-47mRFP	21	23		0		16		0		0	
		% Infected:	0.0%		69.6%		0.0%		Total: 0		
						
vΔRLORF4-47mRFP	28	46		0		14		0		0	
		% Infected:	0.0%		30.4%		0.0%		Total: 0		
vUL47-eGFP	21	31		7		0		0		0	
		% Infected:		22.6%		0.0%		0.0%		Total: 0	
						
vUL47-eGFP	28	50		39		0		0		0	
		% Infected:		78.0%		0.0%		0.0%		Total: 0	
vΔRLORF4-47mRFP +vUL47-eGFP	21	31		9		14		5		2	
		35		3		7		0		0	
		36		18		19		9		4	
		38		0		4		0		0	
		% Infected:		21.4%		31.4%		10.0%		Total: 6	
vΔRLORF4-47mRFP +vUL47-eGFP	28	57		45		16		9		3	
		61		36		28		19		1	
		52		32		24		14		4	
		34		16		8		3		2	
		% Infected:		63.2%		37.3%		22.1%		Total: 10	

a Chickens were inoculated with 2,000 PFU of vΔRLORF4-47mRFP or vUL47-eGFP, or with a mixture of 1,000 PFU of vΔRLORF4-47mRFP and vUL47-eGFP each at 7 days of age.

b Skin samples were collected either 21 or 28 days post-inoculation (p.i.).

c The number of follicles examined for each chicken.

d The number of follicles positive for green fluorescence (vUL47-eGFP replication) and percent follicles infected per group.

### Visual Summary of Coinfection of Feather Follicles, Superinfection Inhibition, and Dual Infection of FFE Cells

**Figure 3:**
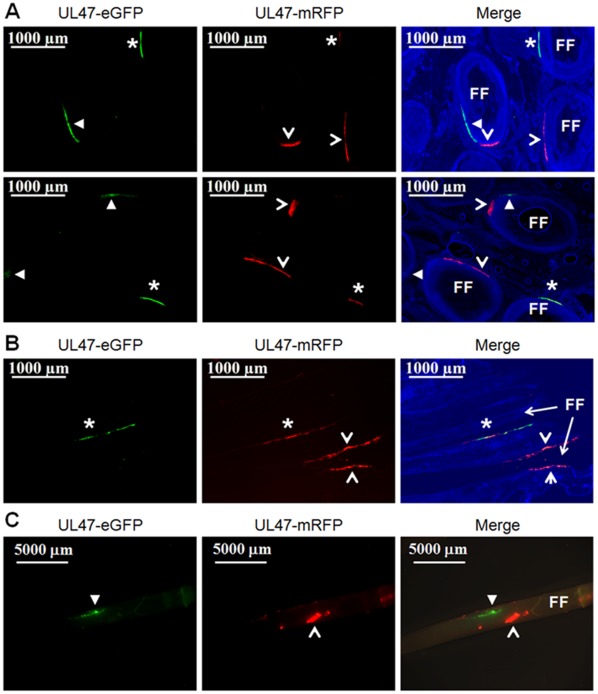
Summary of coinfected feather follicles and dually infected FFE cells at low magnification. Skin/feather tissues were collected from chickens coinfected with vUL47-eGFP and vUL47mRFP (A and B) and sectioned either transversely through the follicle (A) or longitudinally down the follicle (B). Tissues were fixed, stained with Hoechst 33342 to visual nuclei, and examined using an Axio Imager M1 system at ×25 (A and B). Feathers were plucked from the wing of a chicken coinfected with ΔRLORF4-47mRFP and vUL47-eGFP and examined directly for fluorescence using an Olympus SZX-12 Stereoscope at ×5 (C). Arrowheads (◂) indicate regions where only vUL47-eGFP replication is evident, open arrowheads (<) indicate regions where only vUL47-mRFP (A and B) or vΔRLORF4-47mRFP (C) replication are evident, while asterisks (*) indicate regions where both vUL47-eGFP and vUL47-mRFP or vUL47-eGFP and vΔRLORF4-47mRFP are replicating. Feather follicles (FF) are indicated.

Figure 3 shows a summary of representative follicles infected with two different rMDVs at low magnifications using fluorescence microscopy. Figure 3A shows two representative fields of feather follicles transversely cut through the follicles at ×25, Figure 3B shows a representative feather follicle sectioned longitudinally down the follicle at ×25, and Figure 3C shows the subcutaneous portion of a feather follicle plucked from a coinfected chicken at ×5. In some areas of the follicles, only one virus is replicating, indicated by expression of UL47-eGFP or –mRFP alone, while in some areas, dual infection with both viruses is evident (indicated with an asterisk).

**Figure 4:**
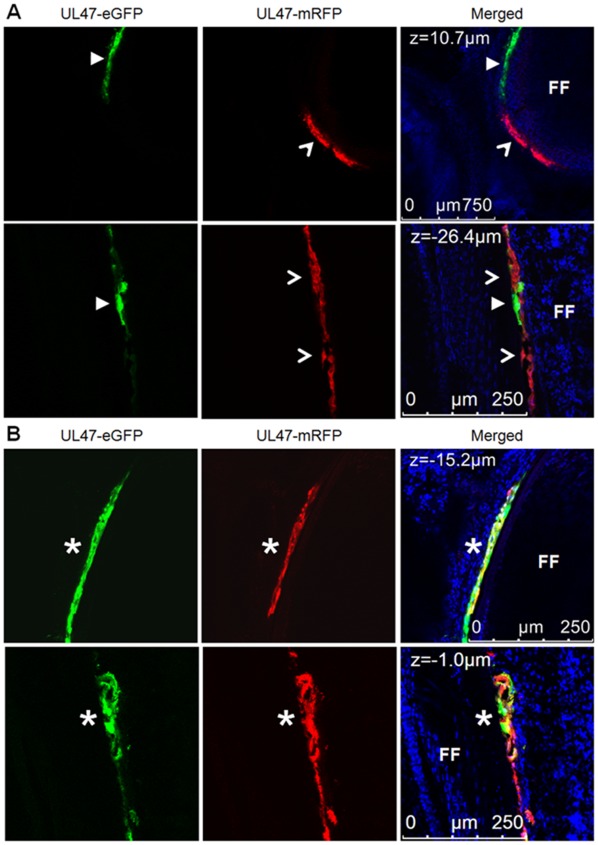
Summary of coinfected feather follicles and dual infected FFE cells at high magnification. Skin/feather tissues were collected from chickens coinfected with vUL47-eGFP and vUL47mRFP and examined using an SP5 LSCM system from Leica Microsystems, Inc. at ×200 (A, top panel) or ×400 (A, bottom panel and B). Hoechst 33342 stain was used to stain nuclei (blue). Single Z-stack images are shown with the depth indicated in the upper left of each image. Arrowheads (◂), open arrowheads (<), and asterisks (*) indicate single and dual infected cells as in Fig. 2. Superinfection inhibition can be seen in (A), while dual infection of FFE cells with both viruses replicating can be seen in (B). Feather follicles (FF) are indicated.

**Figure 5:**
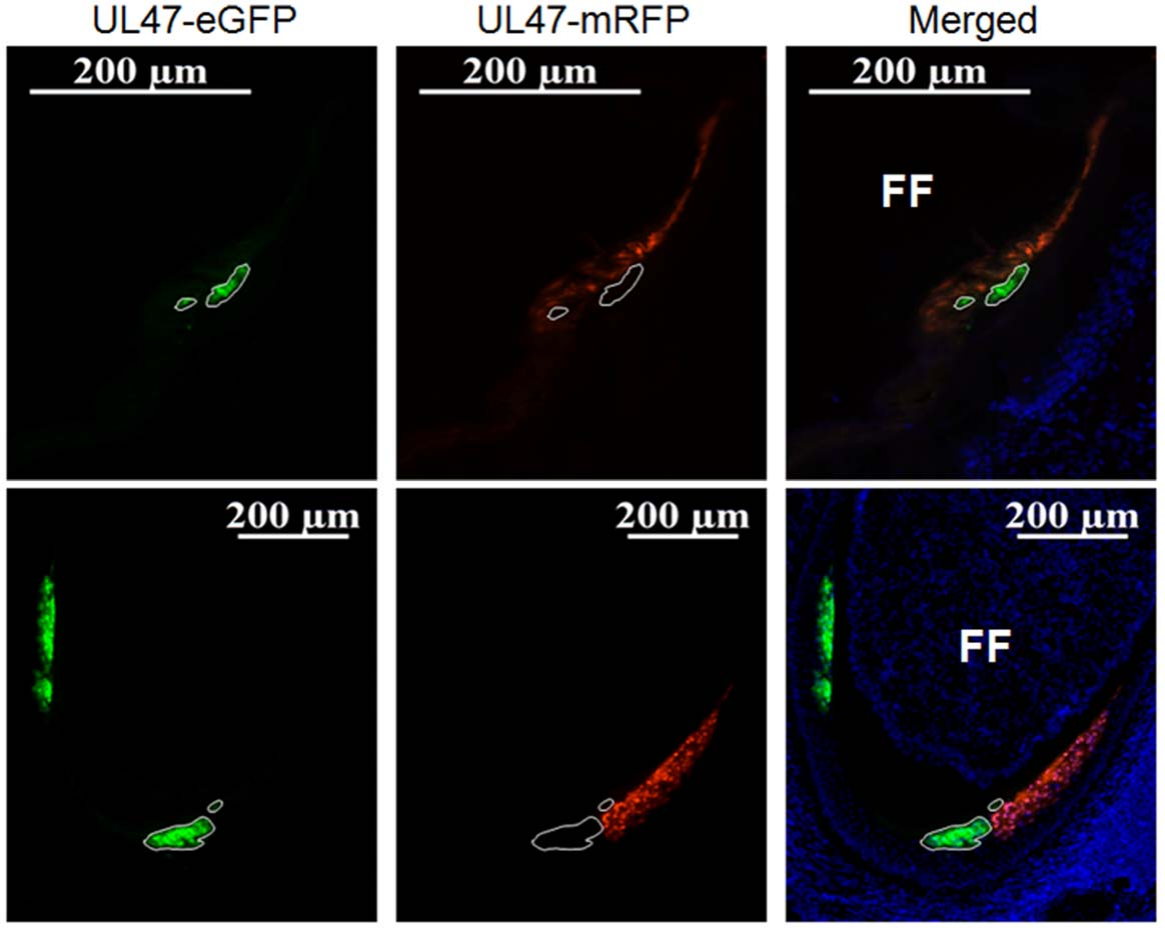
Superinfection inhibition in FFE cells by rMDV. Skin/feather tissues collected from chickens coinfected with vΔRLORF4-mRFP and vUL47-eGFP were examined using an Axio Imager M1 system at ×25. Regions where only vUL47-eGFP is replicating next to cells where only vΔRLORF4-mRFP were traced using Adobe Photoshop and transferred to the accompanying single color image to better show the distinct separation of colors. Feather follicles (FF) are indicated.

Since fluorescence microscopy shows full sample illumination, it was possible that areas in which it appeared that both viruses were infecting the same areas and cells could actually be in different focal planes; therefore, laser scanning confocal microscopy (LSCM) was used to derive Z-stack images. Figure 4 shows representative examples of coinfected feather follicles and dually infected FFE cells during coinfection experiments. Figure 4A shows coinfection of a feather follicle where there is no overlap of replication of each virus in the infected cells. Interestingly, in some areas, in which replication of one virus was predominant, a few cells within the area showed replication of the second virus with no overlap (Fig. 3A, lower panel). Figure 5 highlights these areas.

**Figure 6:**
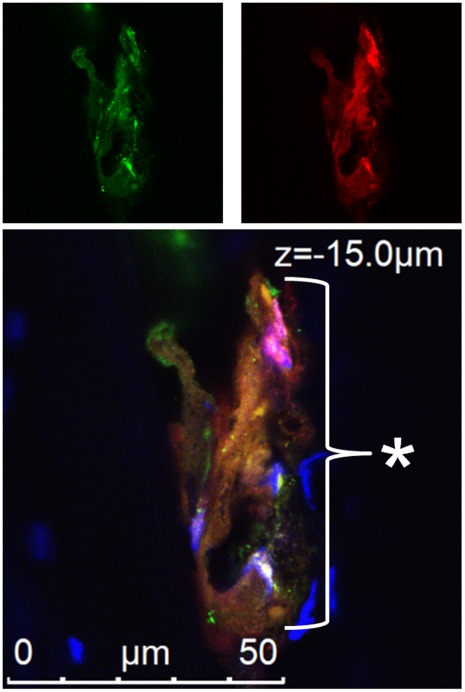
Dual infection of FFE cells with two rMDVs. Skin/feather tissues collected from chickens coinfected with vΔRLORF4-mRFP and vUL47-eGFP in experiment 4 were examined using an SP5 LSCM system from Leica Microsystems, Inc at ×1,890. Cells shown in this Z-stack image are replicating both virus as evidence by expression both UL47-eGFP and UL47-mRFP. The top two panels are green and red images alone, while the bottom panel is the merged image of red and green with Hoechst 33342 (blue) staining of nuclei for contrast. The cells infected with both viruses are indicated with an asterisk (*).

It was also observed that FFE cells could be dually infected with both viruses. This is shown in Figure 4B in which both viruses are replicating within the same cell as indicated by the merging of green and red to make yellow in the Z-stacks shown. Figure 6 shows a single Z-stack image of a dually infected cell. In this Z-stack, a 3–4 cells are seen replicating both red and green viruses (indicated with an asterisk). This image is one Z-stack from multiple Z-stacks shown in Figure S5. These data conclusively show that two very similar alphaherpesviruses can infect the same cells *in vivo* in a natural virus-host model.

## Discussion

It has been previously shown that cells can be infected by two alphaherpesviruses using highly modified viruses in unnatural host models. However, the question still remained whether this occurs in a natural virus-host system. This report utilized the natural herpesvirus model of MDV in chickens to conclusively show, through visualization of fluorescent protein expression by two different rMDVs during *in vivo* infection, that both viruses can dually infect the same cells *in vivo*. These results are important for a number of reasons. For one, this study is the first report directly showing that dual infection with two alphaherpesviruses actively replicating within the same cells occurs in a natural virus-host model, while within the same host, superinfection inhibition can co-occur. Secondly, the fact that chickens are vaccinated against MD with homologous avian alphaherpesviruses that can actively replicate within the same cells in the chicken could potentially lead to exchange of genetic material that could alter virulence of the virus. Thirdly, though there is no direct evidence for dual infection of human skin cells with different VZV strains *in vivo*, recombination between wild-type and vaccine viruses has been reported [39]. These results show direct evidence of dual infection of skin cells by two similar, but distinguishable, alphaherpesvirus that could potentiate exchange of genetic material between virulent and vaccine strains.

The evidence for exchange of genetic elements between different homologous alphaherpesviruses suggests that dual infection of single cells must occur in nature, but until now, it had not been demonstrated that this occurs in a natural virus-host model. It has been shown that rat neurons can be infected with two different PRVs using the well-established rat model for PRV neurovirulence. Banfield *et al.* showed that two attenuated PRV strains, one expressing eGFP and the other expressing mRFP, could infect the same neurons when injected into the anterior chambers of different eyes of rats [17]. Recently, this model was used in intricate transneuronal tracing studies that confirmed dual infection of individual neurons [18]. In both of these studies, the PRV strains used were highly modified from wild-type viruses and the experiments were performed in unnatural hosts. Members of the *Suidae* (true pigs) are the only natural hosts for PRV, although the virus can infect numerous other mammals under experimental conditions [40]. It is suspected that dual infection of cells in pigs occurs since, it has been shown that recombination of PRV vaccines can lead to new strains [12], [13]. Similar evidence of recombination between vaccine and pathogenic strains of ILTV (*GaHV-1*) in chickens has been documented [16], as well as between two mutant *BoHV-1* strains in calves [15]. However, in all previous studies in the natural host, it was never directly demonstrated that genetic exchange or complementation occurred through dual infection of individual cells with two viruses. The data in this report conclusively show that two, actively replicating alphaherpesviruses, can infect the same cells during coinfection in the natural host (Fig. 3, 4, 6, S5).

Though dual infection of individual cells by two rMDVs was clearly evident, it was noteworthy that there also appeared, based on visual observation, to be superinfection inhibition occurring at the cellular level *in vivo* (Fig. 3A, 4A, and 5). Superinfection inhibition, also known as superinfection resistance, has been widely studied *in vitro* [17], [19]–[28], including alphaherpesviruses. Banfield *et al.* [17] used primary rat dorsal root ganglia (DRG) cultures and showed that two different PRVs were able to infect the same DRG *in vitro*, but this only occurred with high frequency (∼100%) when the primary rat DRG cultures were infected simultaneously (coinfection) with both viruses. When one virus was inoculated followed by the second ≥4 h later (superinfection), dual infection of the DRG was very infrequent (∼1%). Thus, their data strongly suggested a significant amount of superinfection inhibition occurs during PRV infection of primary rat DRG neurons *in vitro*. Similar results were observed when examining recombination between two distinguishable *BoHV-1* viruses in Madin-Darby bovine kidney cells *in vitro* [41]. In this study, only simultaneous or superinfection within 4 h led to production of recombinant *BoHV-1,* in which recombination between the two viruses occurred with regularity, whereas infection with one virus and then the other within a time interval of 2 to 8 h allowed the establishment of superinfection inhibition. It has not been demonstrated that superinfection inhibition occurs at the cellular level in the host during infection with homologous alphaherpesviruses, but the data presented in this report suggests that superinfection inhibition occurs during in the natural host during alphaherpesvirus infections (Fig. 3, 4, 5). These studies cannot exclude that FFE cells infected with one virus were not also infected with the second, but the level of fluorescent protein expressed by the second virus was below the level of detection in this system. Further studies are needed to determine if viral genomes of the second virus are present and are the focus of ongoing research.

**Figure 7:**
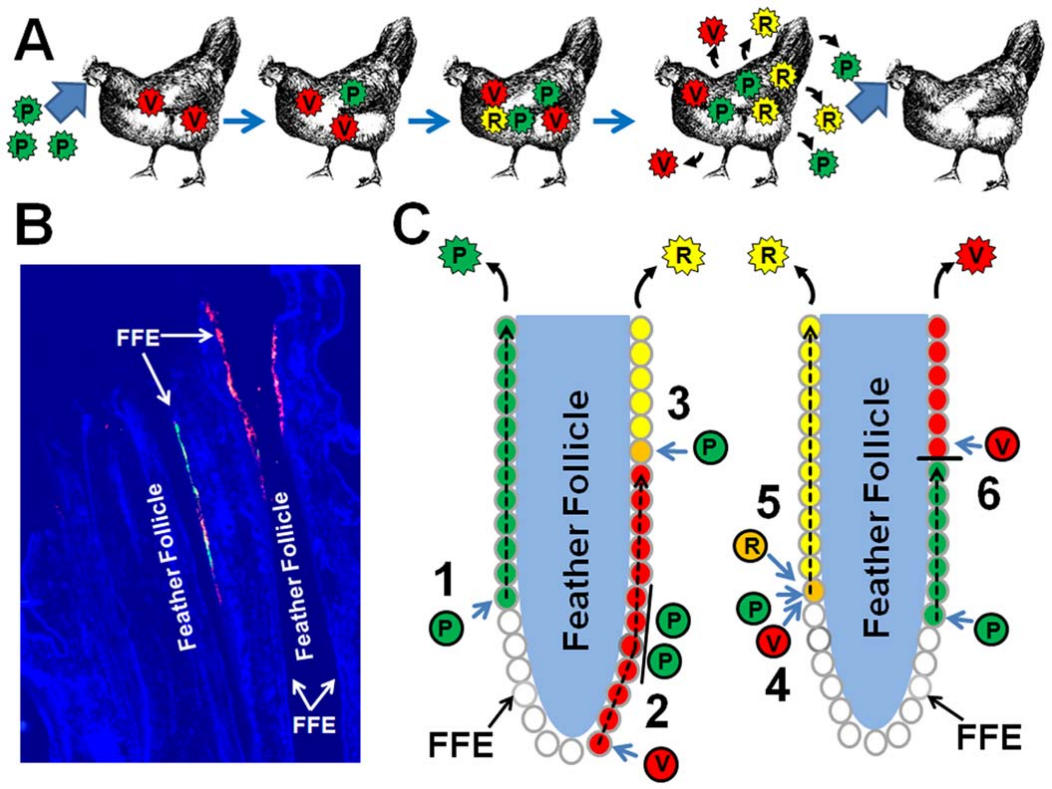
Hypothetical model for coinfection of the host at the organism and cellular level using the chicken as a natural host. (A) During natural infection of chickens previously vaccinated against Marek’s disease, pathogenic (P) MDV enters the chicken through the respiratory route. After entering the host, MDV infects T cells that can then transfer the virus to specialized FFE cells in the skin where infectious virus is produced and shed into the environment and continuing the virus life cycle. Although vaccination with vaccine virus (V) protects chickens from the development of disease, pathogenic MDV can infect and replicate within FFE cells (see below). Should two different pathogenic or a pathogenic and vaccine viruses replicate within the same cell; homologous recombination between the viruses could occur leading to recombinant virus (R). Each virus replicating within the chicken can then be shed into the environment where natural selection will select for traits that benefit the virus. (B) Fluorescence microscopy of feather follicles infected with two similar, but distinguishable viruses expressing either eGFP or mRFP. (C) Schematic diagram of potential scenarios during infection of feather follicles by two MDVs. 1) Pathogenic (shown) or vaccine virus could infect FFE cells and travel along the feather follicle (FF) infecting more FFE cells until the cells and virus are shed from the chicken with no interference from other viruses. 2) Prior infection of FFE cells by the vaccine virus could induce superinfection inhibition within the infected cell, thereby blocking dual infection of the FFE cell by circulating T cells in the skin. 3) Vaccine virus has already infected FFE cells, but pathogenic virus could be transferred from an infected T cell to a newly vaccine infected FFE cell prior to the induction of superinfection inhibition such that both viruses could infect the same FFE cell. This could lead to dual infection of the cell and the generation of recombinant virus (R). 4) Both vaccine and pathogenic viruses circulating within the skin could transfer virus to the same FFE cell at approximately the same time and dually infecting the cell. Again, this could potentiate exchange of genetic material between the viruses generating recombinant virus (R). 5) It is also possible that both vaccine and pathogenic viruses could have dually infected T cells prior to transfer to FFE cells, though the studies presented here do not evaluate this possibility. 6) It is also possible that a virus could be transferred from an infected T cell to FFE cells and as the virus replicates and presumably travels along the FFE tract, encounters cells that have previously been infected with vaccine virus, thereby blocking dual infection. In this scenario, it would be expected the pathogenic virus infects a different group of cells along the FFE tract or replication ceases in these cells. In all scenarios, the final output virus generated and shed would ultimately undergo natural selection for increased virulence.

It is not clear why both superinfection inhibition and dual infection can co-occur within the host, but most likely each event is dependent on the point at which the cell is infected. That is, dual infection is mostly dependent on the cell becoming infected at relatively the same time with both viruses, whereas superinfection inhibition is most likely due to infection of the cell with one virus and after a certain length of time, perhaps >4 h as previous *in vitro* studies might suggest [17], [41], the cell is refractory to infection with the second virus. It is not possible to synchronize infection of the FFE cells with MDV in this natural virus-host system; therefore the time of infection of each FFE cell would most likely be a stochastic process. Figure 7 summarizes potential scenarios in which dual infection of cells and superinfection inhibition would occur in the feather follicles of infected chickens with MDV.

It was interesting that in both superinfection experiments (Tables S1 and S2); active replication of the second virus was not seen in the FFE cells examined. As mentioned above, infection of FFE cells by MDV may be dependent upon circulating infected T cells transferring virus to these cells; therefore the lack of visual evidence of the second inoculated virus replicating in FFE cells could be merely due to a robust immune response to the first inoculated virus. Still, it seemed surprising that no visual evidence of replication of the second virus could be seen in the tissues examined since it is has been established that superinfection of chickens with MDV and MD-vaccine strains does occur with regularity, leading to shedding of both viruses [42], [43]. This would require that infection and replication in the FFE cells must occur in superinfected chickens.

The results presented here are consistent with a recent report evaluating superinfection of chickens with two different virulent MDVs using short- and long-intervals between the first and second inoculations [44]. In this report, Dunn *et al.* also found that in long-interval experiments (13 days between inoculations), evidence of the second inoculated virus was present in only ∼5% of the samples tested compared to ∼55% in short-interval experiments (1 day between inoculations) using highly sensitive pyrosequencing analysis of skin tissues for each virus. However, their method of analysis would not discriminate between latently infected T cells present in the skin and actively replicating virus in FFE cells as is shown in the present study. Though it is difficult to compare the two studies as they used different time points for inoculation and analysis, and different sampling techniques, their data would be consistent with the information presented here and would indicate that a robust immune response against the first inoculated virus most likely explains the lack of replication of the second virus in these experiments. In support of this are data shown in experiment 3 (Table S2) in which the first inoculated virus, highly attenuated vAU5-47mRFP, was barely detected in the feather follicles, yet evidence of the second inoculated virus, virulent vUL47-eGFP, was also not seen in all but one chicken. This chicken was negative for the presence of vAU4-47mRFP in the feather follicles. The parent virus, vAU5 vTR, was previously tested as a vaccine candidate and protected animals against a lethal challenge [37]; therefore this would suggest that a potent immune response to vAU5-47mRFP prevented efficient superinfection by vUL47-eGFP and was not dependent on vAU5-47mRFP infecting feather follicles and inhibiting superinfection with vUL4-eGFP.

It has been presumed that different MDV strains, or related avian herpesviruses like *MeHV-1*, could infect the same feather follicles since superinfection of chickens with different herpesviruses has been documented for many years. However, previous work could not determine whether infected feather follicles were refractory to coinfection or superinfection with a second virus. For example, it is well known that vaccination of chickens with attenuated MDV, non-oncogenic *GaHV-3*, or non-oncogenic *MeHV-1* does not prevent superinfection of the animal, and, subsequently, transmission of pathogenic MDV [3], [31], [45]. There appeared to be at least three scenarios that previous studies examining replication of MDV in feather follicles and superinfection could not discriminate. First, both viruses could readily replicate in the skin, but each virus replicates in separate feather follicles and are refractory to coinfection or superinfection of the same feather follicle (superinfection inhibition of the feather follicle). Second, both viruses could infect the same follicles, but could not dually infect the same cells within that follicle (superinfection inhibition of the cell). Third, both viruses can infect the same cells within a feather follicle (dual infection of the cell).

Based on the data presented here, the first scenario described is incorrect as many feather follicles clearly contain both viruses actively replicating within an individual feather follicle (Fig. 3, 4, 5, 6 and S5). Therefore, there is no evidence for a refractory mechanism of a feather follicle infected with a single virus. However, it does appear that a combination of the remaining two scenarios do co-occur with some regularity. Individual cells or areas infected with a single virus may be refractory to infection and/or replication of the second virus. This is most evident in Figures 3A, 4A, and 5, in which one virus is replicating in a group of cells, while the second virus is replicating in adjacent cells, but replication of both viruses does not overlap. This observation is not unique and could be seen with regularity in coinfected follicles. Clear evidence also exists for dual infection of single cells by both viruses as can be seen in Figures 3A, 4B, 6, and S5. Therefore, within the animal, dual infection of individual cells and superinfection inhibition can co-occur during alphaherpesvirus infection *in vivo*.

The mechanism by which MDV has become more virulent is not known, but homologous recombination with similar avian herpesviruses could play a role. To date, it has not been demonstrated that homologous recombination between MD vaccines and pathogenic MDV has occurred. This is partly due to a lack of efficient tools necessary for such studies; however, major technological advances in the last decade now allow those types of studies. With respect to VZV, it has been shown that recombination between the live varricella vaccine and wild-type VZV occurs during coinfection *in vitro* [46] and has been suggested to occur *in vivo* [47]. Most recently, Breuer and colleagues [39] referred to the emergence of new wild-type/vaccine recombinants by partial-genome sequencing of VZV isolates. The monitoring of VZV genomes since the introduction of vaccines has just begun, but it has been suggested that the frequency of recombinant viruses has mostly likely been underestimated in the past and will increase in coming decades for VZV [48]. The similarities between MDV and VZV with respect to entry into, and exit from, the host are striking as both enter through the respiratory tract, systemically infect T cells that then travel to the skin and transfer virus to epithelial cells, whereupon infectious virus is shed into the environment. In support of these similarities, two homologous genes in both viruses, namely glycoprotein C (MDV UL44, VZV ORF14) and a conserved herpesviral protein kinase (MDV UL13, VZV ORF47) have critical roles during replication in the skin [49], [50] and for transmission [51], [52]. The history of vaccination against MD in chickens with homologous alphaherpesviruses that do not prevent superinfection, and the increasing virulence of MDV over the decades due to vaccination, combined with the definitive proof here that two alphaherpesviruses can dually infect the same cells, should be taken into consideration when designing the next generation of vaccines against MD in chickens and varicella/herpes zoster in humans.

## Materials and Methods

### Ethics Statement

This study was carried out in strict accordance with the recommendations in the Guide for the Care and Use of Laboratory Animals of the National Institutes of Health. The protocol was approved by the Committee on the Ethics of Animal Experiments of Cornell University (permit number 2008-0018). The animal care facilities and programs of Cornell University meet the requirements of the law (89-544, 91-579, 94-276) and NIH regulations on laboratory animals, and are in compliance with the Animal Welfare Act, PL 279. All experimental procedures were in compliance with approval of Cornell University’s Institutional Animal Care and Use Committee and all efforts were made to minimize suffering.

### Cell Cultures and Viruses

Chicken embryo cell cultures were prepared from 10-day-old specific-pathogen-free (SPF) embryos following standard methods [53], and used to reconstitute viruses from pRB-1B DNA. CKC cultures were prepared from 14 day old SPF chickens [53] and used to propagate parental and recombinant viruses. All reconstituted BAC clones were used at ≤5 passages.

### Generation of UL47-tagged Clones

Recombinants with eGFP or mRFP inserted in frame at the C-terminus of UL47 were generated using infectious BAC clones of the RB-1B strain [51] using two-step Red-mediated mutagenesis. vUL47-eGFP was generated and characterized previously [33], while vUL47-mRFP was generated for this report. To generate attenuated rMDV expressing UL47-mRFP, two previously characterized attenuated rMDV BAC clones were used. First, ΔRLORF4, in which both copies of RLORF4 were deleted, has been shown to exhibit attenuated characteristics with increased replication *in vitro* and decreased replication and disease incidence *in vivo* [36]. A second mutant, AU5 vTR, in which both copies of the viral telomerase RNA (vTR) template sequences were mutated (vTR AU5) was also used [37]. This virus replicates like wild-type virus *in vitro*, but is attenuated *in vivo* exhibiting decreased replication levels and abrogation of tumorigenesis.

Briefly, the mRFP-I-*Sce*I-*aphAI* cassette was amplified from pEP-mRFP-in and used for the mutagenesis of each recombinant clones in GS1783 *Escherichia coli* cells as described previously [51]. Mutagenesis primers were as follows: UL47mRFP Forward: 5′**-** gccgtcgaaaccgcccgccgtgagccacaacgggcgaata **tggcctcctccgaggacg -** 3′ and UL47mRFP Reverse: 5′ **-** acatccggagtaaaagtcccgccctcttccctacgtca **caaggcgccggtggagtg -** 3′ (underlined  =  UL47 specific sequences, bold  =  pEP-mRFP-in specific sequences). All recombinant clones were confirmed by restriction fragment length polymorphism (RFLP), PCR, and DNA sequencing.

rMDVs were reconstituted by transfecting chick embryo cell cultures with purified BAC DNA using the CaPO_4_ precipitation method [54] with a plasmid expressing the *Cre* enzyme (pCAGGS-NLS/*Cre*) for excision of mini-F sequences using loxP sites and screened as previously described [51]. See Figure 1 and Table 1 for more detailed description of the viruses used in this study.

### Measurement of Plaque Areas

Plaque areas were measured exactly as previously described [33]. Plaque areas were measured using ImageJ [55] version 1.41o software (rsb.info.nih.gov/ij) and means were determined for each virus. Significant differences in mean plaque areas were determined using Student’s *t* tests.

### Fluorescence Microscopy

CKC cultures were infected with vUL47-eGFP, vUL47-mRFP, vΔRLORF4-47mRFP, or vAU5-47mRFP on sterile glass coverslips in 24-well dishes at 50 PFU per well. At 5 days p.i., cells were fixed with PFA buffer (2% paraformaldehyde, 0.1% Triton X-100) for 15 min and then washed twice with phosphate-buffered saline. Skin/feather tissues were collected from the ventral feather tracts of rMDV-infected chickens in 15×10 mm sections and snap-frozen in Tissue Tek®-optimal cutting temperature (OCT) compound (Sankura® Finetek, Torrance, CA) and stored at -80°C until sectioned. Eight µm sections were affixed to Superfrost/Plus slides (Fisher Scientific, Pittsburgh, PA) and fixed as described above. Primary flight feathers were plucked from the left wing of rMDV-infected chickens and immediately viewed with a stereoscope.

Infected CKC cultures and cryosectioned tissues used for antigen detection were blocked in 10% neonatal calf serum, stained with monoclonal antibody O11 directed against the early lytic MDV protein pp38 [56] or chicken anti-MDV antiserum [36]. Goat anti-mouse IgG-Alexa Fluor® 488 or 568 and goat anti-chicken IgY-Alexa Fluor® 488 or 568 (Molecular Probes, Eugene, OR) were used as secondary antibodies. Hoechst 33342 (2 µg/ml, Molecular Probes) was used to visualize nuclei. An Axio Imager M1 system with AxioVision software (Carl Zeiss, Inc., Thornwood, NY) or a Leica Microsystems, Inc. (Buffalo Grove, IL) SP5 LSCM system was used to analyze stained coverslips or slides. The following fluorescent dyes were excited and emissions detected with the SP5 LCSM as follows: Hoechst 33343 excited at 405 nm with a UV diode laser and detected at 417–462 nm; eGFP and Alexa Fluor® 488 excited with an Argon laser (458 nm laser line) and detected at 498-550 nm; mRFP and Alexa Fluor® 568 excited at 561 nm with a Diode-pump Solid State (DPSS) laser and detected at 566-632 nm. The Olympus SZX-12 Stereoscope (kindly provided by the Plant Cell Imaging Center at The Boyce Thompson Institute for Plant Research, Ithaca, NY) was used for analysis of whole skin/feather and plucked feathers at low magnification. The long pass (LP) Green (excitation 470 nm, emission LP 500 nm) and Red (excitation 540 nm, emission 605 nm) filter cubes were used to detect eGFP and mRFP, respectively. All images were compiled using Adobe® Photoshop® CS2 version 9.0.2.

### Animal Studies

SPF P2a (MHC: *B^19^B^19^*) chickens were obtained from departmental flocks and housed in isolation units. Water and food were provided *ad libitum*. Chickens were inoculated intra-abdominally with 1,000 or 2,000 PFU of each virus at various times dependent on the experiment. Virus inocula consisted of rMDV-infected CKC cultures. For coinfection experiments, virus inocula were mixed prior to injection into chickens. Chickens were assigned to inoculation groups using a randomization table.

## Supporting Information

Figure S1 **Plaque area assays of rMDV**. Plaque areas were measured for viruses reconstituted from rParental (vParental), rUL47-eGFP (vUL47-eGFP), rUL47-mRFP (vUL47-mRFP), rΔRLORF4 (vΔRLORF4), rΔRLORF4-47mRFP (vΔRLORF4-47mRFP), rAU5 (vAU5), and rAU5-47mRFP (vAU5-47mRFP). Error bars represent standard error of the means for each group (n  = 30). Both ΔRLORF4 viruses induced plaques that were significantly different (vΔRLORF4, *P*  = 7.3×10^−6^; vΔRLORF4-47mRFP, *P*  = 2.4×10^−5^) from vParental using Student’s *t* tests and are indicated with an asterisk (*).
(TIF)

Figure S2 **Expression of UL47-eGFP and UL47-mRFP fusion proteins** ***in vitro*** **.** CKC cultures were infected with vParental, vUL47-eGFP, vUL47-mRFP, vΔRLORF4-47mRFP, or vAU5-47mRFP on glass coverslips and then fixed at 4 days p.i. An anti-MDV chicken antibody was used to identify overall MDV antigen expression with goat anti-chicken IgG-Alexa Fluor® 568 (A) or 488 (B) secondary antibody, and Hoechst 33342 was used to identify nuclei (blue). For each plaque, the same parameters (lasers, excitation/emission wavelengths, time of exposure, magnification, etc.) were used to compare the fluorescence intensities. Merged images contain all three fluorescent channels. Images were recorded at ×100 magnification using an Axio Imager M1 system with AxioVision software and compiled using Adobe Photoshop.
(TIF)

Figure S3 **Tumor induction by rMDVs.** Tumor incidence was determined over the course of 56 days in chickens infected with vUL47-eGFP, vUL47-mRFP, vΔRLORF4-47mRFP, or vAU5-47mRFP (n  = 18 to 20). Chickens were evaluated daily for clinical signs of MD, euthanized when symptoms were apparent, and necropsies were performed to identify tumor lesions. As expected, both vUL47-eGFP and -mRFP were highly virulent [33], while vΔRLORF4-47mRFP was highly attenuated and vAU5-47mRFP was completely non-oncogenic, as previously described for both [36], [37].
(TIF)

Figure S4 **Experimental design for coinfection and superinfection of chickens with different rMDVs.** Four experiments were designed to coinfect (A and D) or superinfect (B and C) chickens with different fluorescently-tagged UL47 rMDVs.
(TIF)

Figure S5 **Multiple Z-stack images of FFE cells dually infected with two rMDVs.** A total of 50 Z-stack images were collected from this tissue to view the replication of vΔRLORF4-47mRFP and vUL47-eGFP through an 8 µm section. Only shown are every ten Z-stack images, excluding the last image that was negative for both colors. Both viruses can be seen replicating in the same cells throughout the Z-stacks.
(TIF)

Table S1 **Superinfection with virulent viruses (Experiment 2) to observe dual infection of feather follicle epithelial cells.**
(DOC)

Table S2 **Superinfection with attenuated and virulent viruses (Experiment 3) to observe dual infection of feather follicle epithelial cells.**
(DOC)
