# Supplementary figures and images for: Dual Infection and Superinfection Inhibition of Epithelial Skin Cells by Two Alphaherpesviruses Co-Occur in the Natural Host
Source: PLoS One. 2012 May 21;7(5):e37428. doi: 10.1371/journal.pone.0037428 (PMC3357410; doi:10.1371/journal.pone.0037428)

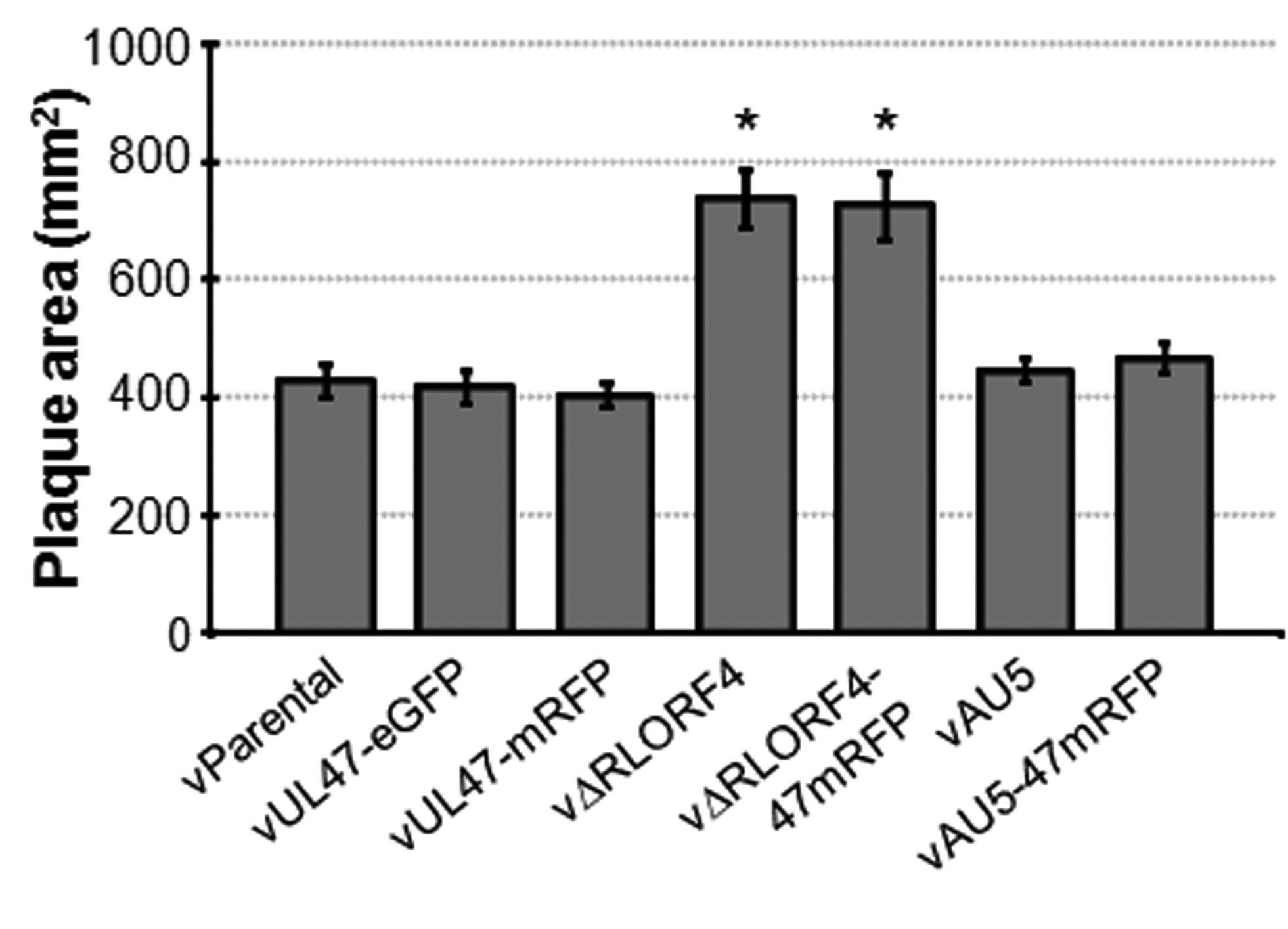

Supplement: Figure S1 — Plaque area assays of rMDV. Plaque areas were measured for viruses reconstituted from rParental (vParental), rUL47-eGFP (vUL47-eGFP), rUL47-mRFP (vUL47-mRFP), rΔRLORF4 (vΔRLORF4), rΔRLORF4-47mRFP (vΔRLORF4-47mRFP), rAU5 (vAU5), and rAU5-47mRFP (vAU5-47mRFP). Error bars represent standard error of the means for each group (n = 30). Both ΔRLORF4 viruses induced plaques that were significantly different (vΔRLORF4, P = 7.3×10^−6^; vΔRLORF4-47mRFP, P = 2.4×10^−5^) from vParental using Student’s t tests and are indicated with an asterisk (*). (TIF) [file pone.0037428.s001.tif]

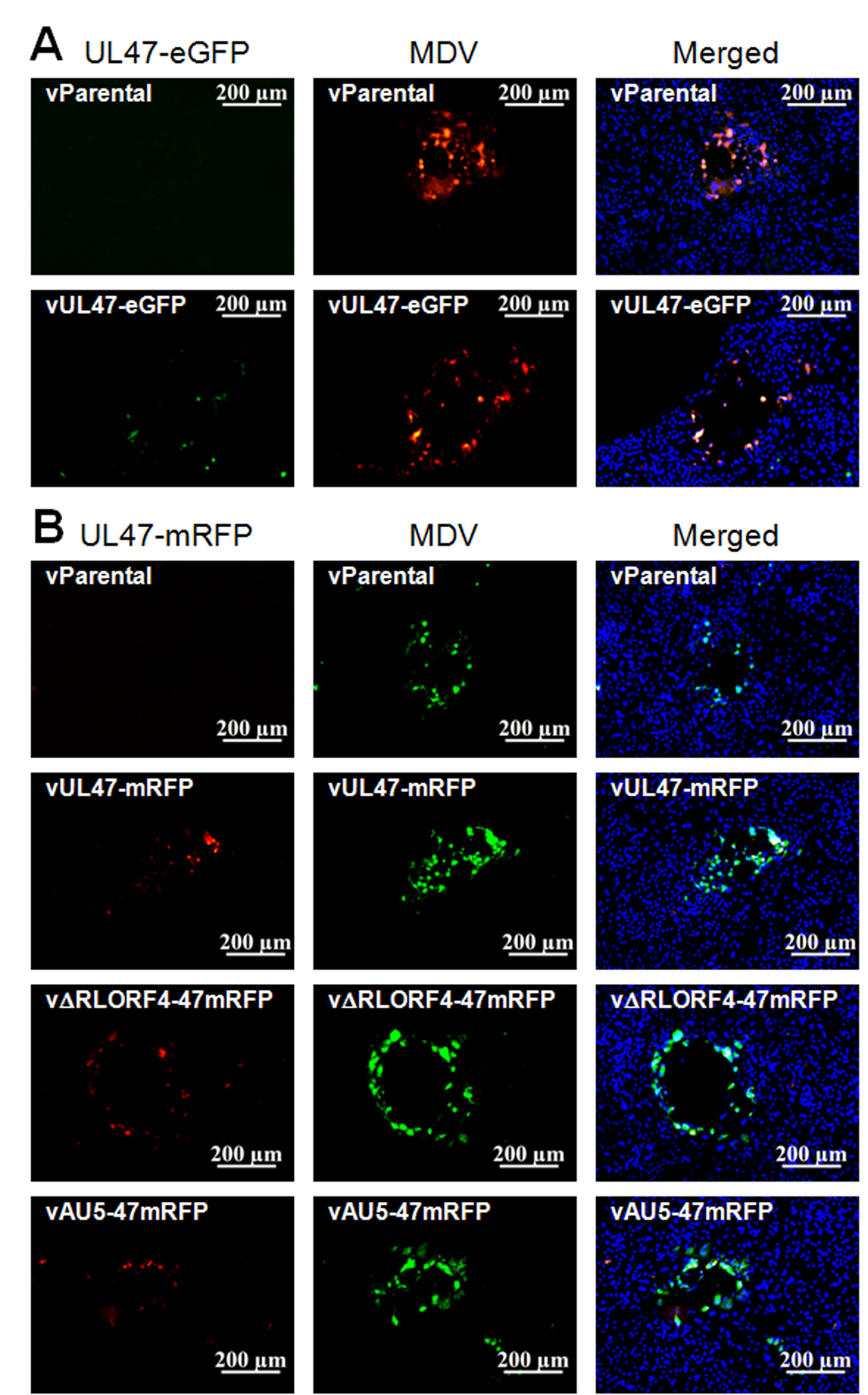

Supplement: Figure S2 — Expression of UL47-eGFP and UL47-mRFP fusion proteins in vitro . CKC cultures were infected with vParental, vUL47-eGFP, vUL47-mRFP, vΔRLORF4-47mRFP, or vAU5-47mRFP on glass coverslips and then fixed at 4 days p.i. An anti-MDV chicken antibody was used to identify overall MDV antigen expression with goat anti-chicken IgG-Alexa Fluor® 568 (A) or 488 (B) secondary antibody, and Hoechst 33342 was used to identify nuclei (blue). For each plaque, the same parameters (lasers, excitation/emission wavelengths, time of exposure, magnification, etc.) were used to compare the fluorescence intensities. Merged images contain all three fluorescent channels. Images were recorded at ×100 magnification using an Axio Imager M1 system with AxioVision software and compiled using Adobe Photoshop. (TIF) [file pone.0037428.s002.tif]

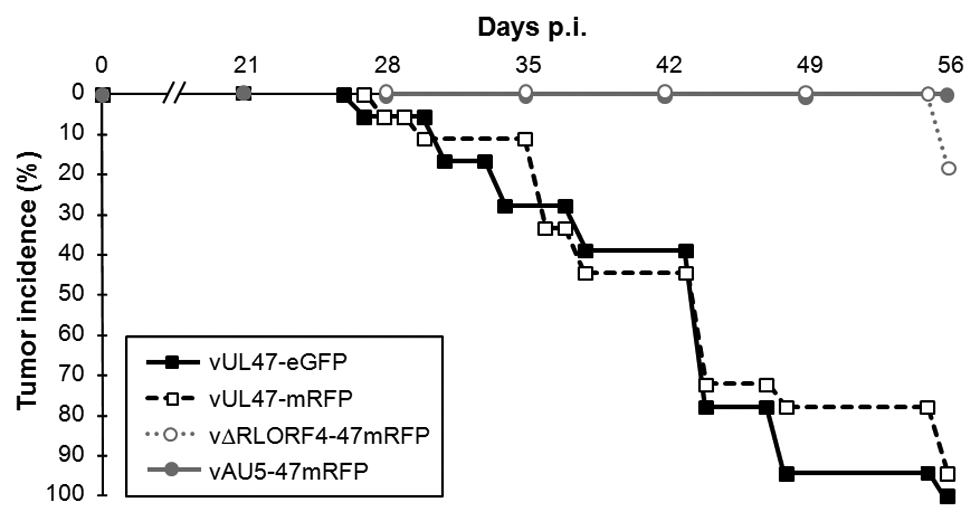

Supplement: Figure S3 — Tumor induction by rMDVs. Tumor incidence was determined over the course of 56 days in chickens infected with vUL47-eGFP, vUL47-mRFP, vΔRLORF4-47mRFP, or vAU5-47mRFP (n = 18 to 20). Chickens were evaluated daily for clinical signs of MD, euthanized when symptoms were apparent, and necropsies were performed to identify tumor lesions. As expected, both vUL47-eGFP and -mRFP were highly virulent [33], while vΔRLORF4-47mRFP was highly attenuated and vAU5-47mRFP was completely non-oncogenic, as previously described for both [36], [37]. (TIF) [file pone.0037428.s003.tif]

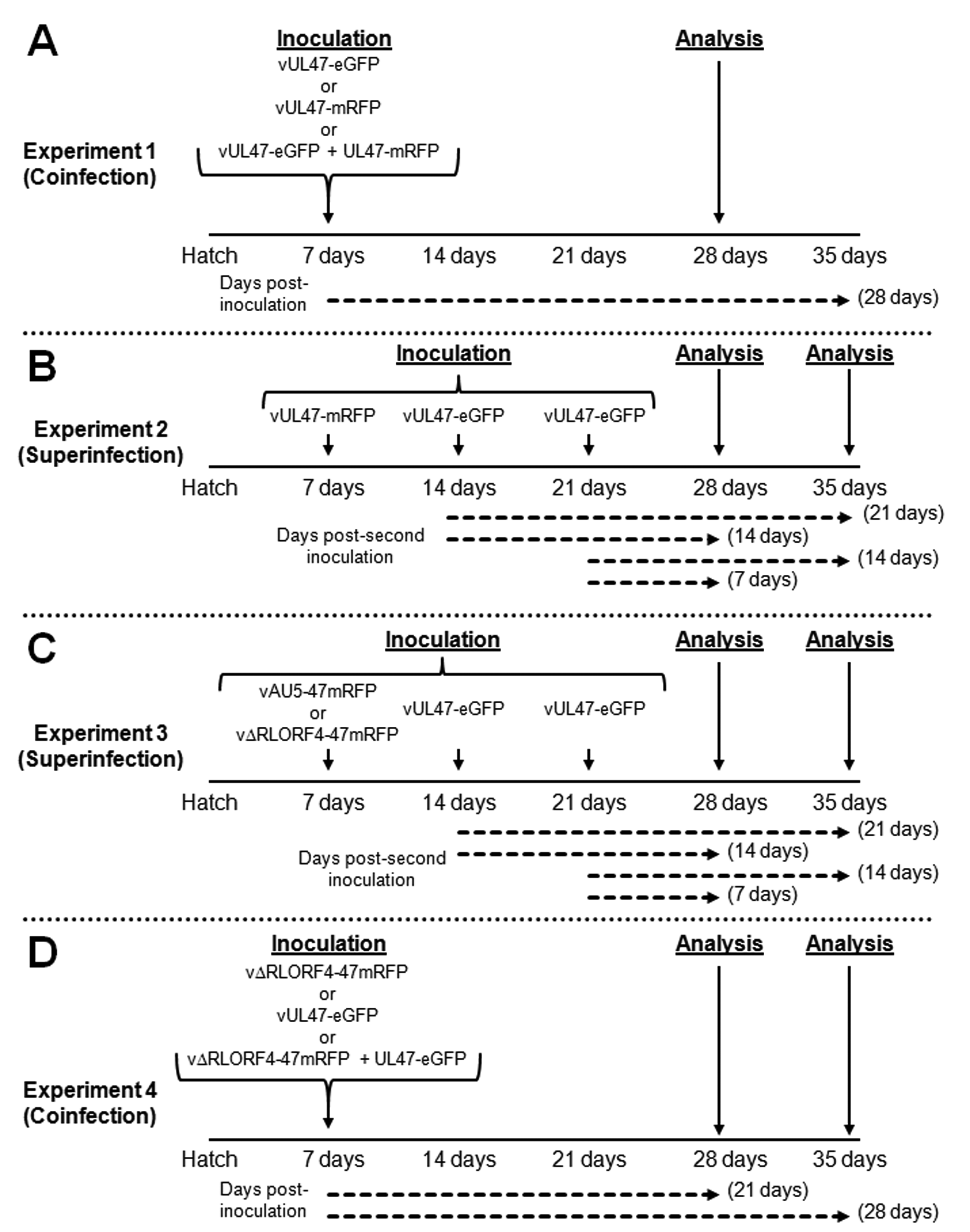

Supplement: Figure S4 — Experimental design for coinfection and superinfection of chickens with different rMDVs. Four experiments were designed to coinfect (A and D) or superinfect (B and C) chickens with different fluorescently-tagged UL47 rMDVs. (TIF) [file pone.0037428.s004.tif]

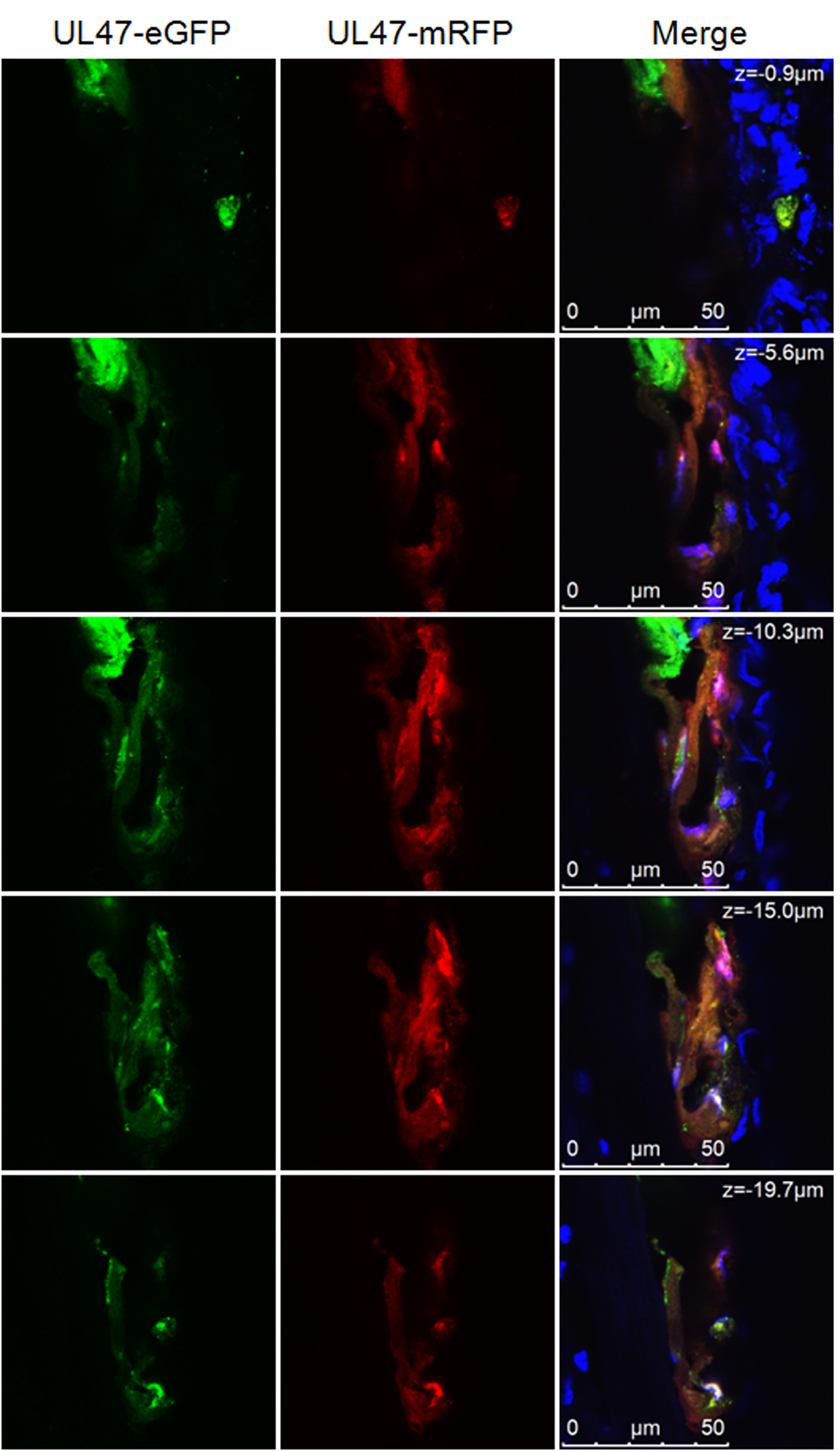

Supplement: Figure S5 — Multiple Z-stack images of FFE cells dually infected with two rMDVs. A total of 50 Z-stack images were collected from this tissue to view the replication of vΔRLORF4-47mRFP and vUL47-eGFP through an 8 µm section. Only shown are every ten Z-stack images, excluding the last image that was negative for both colors. Both viruses can be seen replicating in the same cells throughout the Z-stacks. (TIF) [file pone.0037428.s005.tif]
