# Supplementary material for: Dual Infection and Superinfection Inhibition of Epithelial Skin Cells by Two Alphaherpesviruses Co-Occur in the Natural Host
Source: PLoS One. 2012 May 21;7(5):e37428. doi: 10.1371/journal.pone.0037428 (PMC3357410; doi:10.1371/journal.pone.0037428)
Supplement: Table S1 — Superinfection with virulent viruses (Experiment 2) to observe dual infection of feather follicle epithelial cells. (DOC) [file pone.0037428.s006.doc]

**Table S1. Superinfection with virulent viruses (Experiment 2).**

| 1° inoculation*^a^* |  | 2° inoculation (days p.p.i.)*^b^* |  | Analysis (days p.s.i.)*^c^* |  | Follicles (n)*^d^* |  | Green*^e^* |  | Red*^f^* |  | Coinfection Follicle*^g^* |  | Dual infection Cell*^h^* |
| --- | --- | --- | --- | --- | --- | --- | --- | --- | --- | --- | --- | --- | --- | --- |
|  |  |  |  |  |  |  |  |  |  |  |  |  |  |  |
| None |  | vUL47-eGFP (7) |  | 21 |  | 8 |  | 3 |  | 0 |  | 0 |  | 0 |
|  |  |  |  |  |  | % Infected: |  | 37.5% |  | 0.0% |  | 0.0% |  | Total: 0 |
|  |  |  |  |  |  |  |  |  |  |  |  |  |  |  |
| vUL47- mRFP |  | vUL47-eGFP (7) |  | 14 |  | 31 |  | 0 |  | 10 |  | 0 |  | 0 |
|  |  |  |  |  |  | 14 |  | 0 |  | 3 |  | 0 |  | 0 |
|  |  |  |  |  |  | 14 |  | 0 |  | 2 |  | 0 |  | 0 |
|  |  |  |  |  |  | 29 |  | 0 |  | 13 |  | 0 |  | 0 |
|  |  |  |  |  |  | 21 |  | 0 |  | 7 |  | 0 |  | 0 |
|  |  |  |  |  |  | 26 |  | 0 |  | 7 |  | 0 |  | 0 |
|  |  |  |  |  |  | % Infected: | | 0.0% |  | 31.1% |  | 0.0% |  | Total: 0 |
|  |  |  |  |  |  |  |  |  |  |  |  |  |  |  |
| vUL47- mRFP |  | vUL47-eGFP (7) |  | 21 |  | 26 |  | 0 |  | 14 |  | 0 |  | 0 |
|  |  |  |  |  |  | 14 |  | 0 |  | 5 |  | 0 |  | 0 |
|  |  |  |  |  |  | 13 |  | 0 |  | 1 |  | 0 |  | 0 |
|  |  |  |  |  |  | 17 |  | 0 |  | 9 |  | 0 |  | 0 |
|  |  |  |  |  |  | 17 |  | 0 |  | 4 |  | 0 |  | 0 |
|  |  |  |  |  |  | 13 |  | 0 |  | 2 |  | 0 |  | 0 |
|  |  |  |  |  |  | 14 |  | 0 |  | 9 |  | 0 |  | 0 |
|  |  |  |  |  |  | % Infected: | | 0.0% |  | 38.6% |  | 0.0% |  | Total: 0 |
|  |  |  |  |  |  |  |  |  |  |  |  |  |  |  |
| vUL47- mRFP |  | vUL47-eGFP (14) |  | 7 |  | 23 |  | 0 |  | 6 |  | 0 |  | 0 |
|  |  |  |  |  |  | 35 |  | 0 |  | 13 |  | 0 |  | 0 |
|  |  |  |  |  |  | 22 |  | 0 |  | 14 |  | 0 |  | 0 |
|  |  |  |  |  |  | 23 |  | 0 |  | 8 |  | 0 |  | 0 |
|  |  |  |  |  |  | 18 |  | 0 |  | 3 |  | 0 |  | 0 |
|  |  |  |  |  |  | % Infected: | | 0.0% |  | 36.4% |  | 0.0% |  | Total: 0 |
|  |  |  |  |  |  |  |  |  |  |  |  |  |  |  |
| vUL47- mRFP |  | vUL47-eGFP (14) |  | 14 |  | 12 |  | 0 |  | 5 |  | 0 |  | 0 |
|  |  |  |  |  |  | 12 |  | 0 |  | 4 |  | 0 |  | 0 |
|  |  |  |  |  |  | 13 |  | 0 |  | 3 |  | 0 |  | 0 |
|  |  |  |  |  |  | 11 |  | 0 |  | 5 |  | 0 |  | 0 |
|  |  |  |  |  |  | % Infected: | | 0.0% |  | 35.4% |  | 0.0% |  | Total: 0 |
|  |  |  |  |  | | | |  |  |  |  |  |  |  |

*^a^*Chickens were inoculated with 2,000 PFU of vUL47-mRFP at 7 days of age.

*^b^*Chickens were inoculated with 2,000 PFU of vUL47-eGFP at 7 or 14 days post primary inoculation (p.p.i).

*^c^*Skin samples were collected at 7, 14, or 21 days post secondary inoculation (p.s.i.) to analyze replication of primary and secondary viruses.

*^d^*The number of follicles examined for each chicken.

*^e^*The number of follicles positive for green fluorescence (vUL47-eGFP replication) and percent follicles infected per group.

*^f^*The number of follicles positive for red fluorescence (vUL47-mRFP replication) and percent follicles infected per group.

*^g^*The number of follicles positive for both green and red fluorescence and percent follicles infected with both viruses per group.

*^h^*The number of areas within follicles that were positive for both green and red fluorescence and the total per group.
