# Supplementary material for: Dual Infection and Superinfection Inhibition of Epithelial Skin Cells by Two Alphaherpesviruses Co-Occur in the Natural Host
Source: PLoS One. 2012 May 21;7(5):e37428. doi: 10.1371/journal.pone.0037428 (PMC3357410; doi:10.1371/journal.pone.0037428)
Supplement: Table S2 — Superinfection with attenuated and virulent viruses (Experiment 3) to observe dual infection of feather follicle epithelial cells. (DOC) [file pone.0037428.s007.doc]

**Table S2. Superinfection with attenuated and virulent viruses (Experiment 3).**

| 1° inoculation*^a^* |  | 2° inoculation (days p.p.i.)*^b^* |  | Analysis (days p.s.i.)*^c^* |  | Follicles (n)*^d^* |  | Green*^e^* |  | Red*^f^* |  | Coinfection Follicle*^g^* |  | Dual infection Cell*^h^* |
| --- | --- | --- | --- | --- | --- | --- | --- | --- | --- | --- | --- | --- | --- | --- |
|  |  |  |  |  |  |  |  |  |  |  |  |  |  |  |
| None |  | vUL47-eGFP (7) |  | 14 |  | 49 |  | 29 |  | 0 |  | 0 |  | 0 |
|  |  |  |  |  |  | % Infected: |  | 59.2% |  | 0.0% |  | 0.0% |  | Total: 0 |
|  |  |  |  |  |  |  |  |  |  |  |  |  |  |  |
| vΔRLORF4-47mRFP |  | vUL47-eGFP (7) |  | 14 |  | 103 |  | 0 |  | 48 |  | 0 |  | 0 |
|  |  |  |  |  |  | 86 |  | 0 |  | 38 |  | 0 |  | 0 |
|  |  |  |  |  |  | 91 |  | 0 |  | 26 |  | 0 |  | 0 |
|  |  |  |  |  |  | 115 |  | 0 |  | 9 |  | 0 |  | 0 |
|  |  |  |  |  |  | 114 |  | 0 |  | 78 |  | 0 |  | 0 |
|  |  |  |  |  |  | % Infected: |  | 0.0% |  | 39.1% |  | 0.0% |  | Total: 0 |
|  |  |  |  |  |  |  |  |  |  |  |  |  |  |  |
| vΔRLORF4-47mRFP |  | vUL47-eGFP (7) |  | 21 |  | 75 |  | 0 |  | 37 |  | 0 |  | 0 |
|  |  |  |  |  |  | 53 |  | 0 |  | 32 |  | 0 |  | 0 |
|  |  |  |  |  |  | 64 |  | 0 |  | 45 |  | 0 |  | 0 |
|  |  |  |  |  |  | 75 |  | 0 |  | 29 |  | 0 |  | 0 |
|  |  |  |  |  |  | 38 |  | 0 |  | 24 |  | 0 |  | 0 |
|  |  |  |  |  |  | % Infected: |  | 0.0% |  | 54.8% |  | 0.0% |  | Total: 0 |
|  |  |  |  |  |  |  |  |  |  |  |  |  |  |  |
| vΔRLORF4-47mRFP |  | vUL47-eGFP (14) |  | 7 |  | 102 |  | 0 |  | 53 |  | 0 |  | 0 |
|  |  |  |  |  |  | 122 |  | 0 |  | 46 |  | 0 |  | 0 |
|  |  |  |  |  |  | 83 |  | 0 |  | 61 |  | 0 |  | 0 |
|  |  |  |  |  |  | 94 |  | 0 |  | 47 |  | 0 |  | 0 |
|  |  |  |  |  |  | 80 |  | 0 |  | 41 |  | 0 |  | 0 |
|  |  |  |  |  |  | % Infected: |  | 0.0% |  | 51.8% |  | 0.0% |  | Total: 0 |
|  |  |  |  |  |  |  |  |  |  |  |  |  |  |  |
| vΔRLORF4-47mRFP |  | vUL47-eGFP (14) |  | 14 |  | 65 |  | 0 |  | 49 |  | 0 |  | 0 |
|  |  |  |  |  |  | 54 |  | 0 |  | 50 |  | 0 |  | 0 |
|  |  |  |  |  |  | 75 |  | 0 |  | 56 |  | 0 |  | 0 |
|  |  |  |  |  |  | 74 |  | 0 |  | 39 |  | 0 |  | 0 |
|  |  |  |  |  |  | 62 |  | 0 |  | 54 |  | 0 |  | 0 |
|  |  |  |  |  |  | % Infected: |  | 0.0% |  | 75.2% |  | 0.0% |  | Total: 0 |
|  |  |  |  |  |  |  |  |  |  |  |  |  |  |  |
| vAU5- 47mRFP |  | vUL47-eGFP (7) |  | 14 |  | 101 |  | 0 |  | 0 |  | 0 |  | 0 |
|  |  |  |  |  |  | 91 |  | 0 |  | 0 |  | 0 |  | 0 |
|  |  |  |  |  |  | 106 |  | 0 |  | 0 |  | 0 |  | 0 |
|  |  |  |  |  |  | 120 |  | 0 |  | 1 |  | 0 |  | 0 |
|  |  |  |  |  |  | 113 |  | 0 |  | 0 |  | 0 |  | 0 |
|  |  |  |  |  |  | % Infected: |  | 0.0% |  | 0.2% |  | 0.0% |  | Total: 0 |
|  |  |  |  |  |  |  |  |  |  |  |  |  |  |  |
| vAU5- 47mRFP |  | vUL47-eGFP (7) |  | 21 |  | 65 |  | 0 |  | 0 |  | 0 |  | 0 |
|  |  |  |  |  |  | 92 |  | 0 |  | 1 |  | 0 |  | 0 |
|  |  |  |  |  |  | 58 |  | 1 |  | 0 |  | 0 |  | 0 |
|  |  |  |  |  |  | 62 |  | 0 |  | 0 |  | 0 |  | 0 |
|  |  |  |  |  |  | 54 |  | 18 |  | 0 |  | 0 |  | 0 |
|  |  |  |  |  |  | % Infected: |  | 5.7% |  | 0.3% |  | 0.0% |  | Total: 0 |
|  |  |  |  |  |  |  |  |  |  |  |  |  |  |  |
| vAU5- 47mRFP |  | vUL47-eGFP (14) |  | 7 |  | 83 |  | 0 |  | 0 |  | 0 |  | 0 |
|  |  |  |  |  |  | 71 |  | 0 |  | 0 |  | 0 |  | 0 |
|  |  |  |  |  |  | 98 |  | 0 |  | 0 |  | 0 |  | 0 |
|  |  |  |  |  |  | 98 |  | 0 |  | 0 |  | 0 |  | 0 |
|  |  |  |  |  |  | 103 |  | 0 |  | 0 |  | 0 |  | 0 |
|  |  |  |  |  |  | % Infected: |  | 0.0% |  | 0.0% |  | 0.0% |  | Total: 0 |
|  |  |  |  |  |  |  |  |  |  |  |  |  |  |  |
| vAU5- 47mRFP |  | vUL47-eGFP (14) |  | 14 |  | 58 |  | 0 |  | 0 |  | 0 |  | 0 |
|  |  |  |  |  |  | 50 |  | 0 |  | 0 |  | 0 |  | 0 |
|  |  |  |  |  |  | 51 |  | 0 |  | 0 |  | 0 |  | 0 |
|  |  |  |  |  |  | 59 |  | 0 |  | 0 |  | 0 |  | 0 |
|  |  |  |  |  |  | 51 |  | 0 |  | 0 |  | 0 |  | 0 |
|  |  |  |  |  |  | % Infected: |  | 0.0% |  | 0.0% |  | 0.0% |  | Total: 0 |
|  |  |  |  |  | | | |  |  |  |  |  |  |  |

*^a^*Seven-day old chickens were inoculated with 2,000 PFU of vΔRLORF4-47mRFP or vAU5-47mRFP.

*^b^*Chickens were inoculated with 2,000 PFU of vUL47-eGFP at 7 or 14 days post primary inoculation (p.p.i).

*^c^*Skin samples were collected at 7, 14, or 21 days post secondary inoculation (p.s.i.) to analyze replication of primary and secondary viruses.

*^d^*The number of follicles examined for each chicken.

*^e^*The number of follicles positive for green fluorescence (vUL47-eGFP replication) and percent follicles infected per group.

*^f^*The number of follicles positive for red fluorescence (vΔRLORF4-47mRFP or vAU5-47mRFP replication) and percent follicles infected per group.

*^g^*The number of follicles positive for both green and red fluorescence and percent follicles infected with both viruses per group.

*^h^*The number of areas within follicles that were positive for both green and red fluorescence and the total per group.
